# Supplementary material for: Co-Expression Network Analysis of Spleen Transcriptome in Rock Bream (Oplegnathus fasciatus) Naturally Infected with Rock Bream Iridovirus (RBIV)
Source: Int J Mol Sci. 2020 Mar 2;21(5):1707. doi: 10.3390/ijms21051707 (PMC7084886; doi:10.3390/ijms21051707)

Figure S1. (A) PCA plot of 35,861 unigenes from 25 samples. Dot colors represent analyzed groups: Group 0C (black), 0H (orange), 0MH (red), 3C (blue), and 3L (green). (B) Clustering dendrogram of 25 samples and heatmap with other clinical traits. Clustering was conducted based on FPKM value from 25 samples by calculating correlation adjacency with unsigned type. Color intensity represents relative value of each trait.

(A)

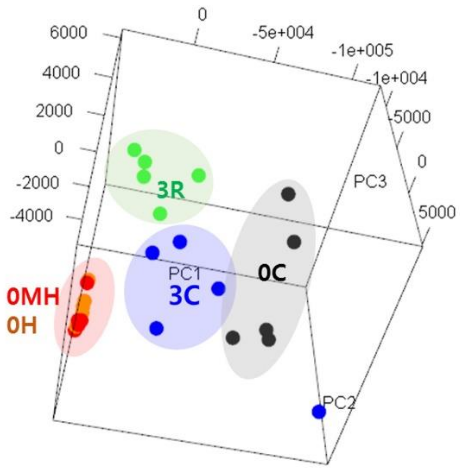

(B)

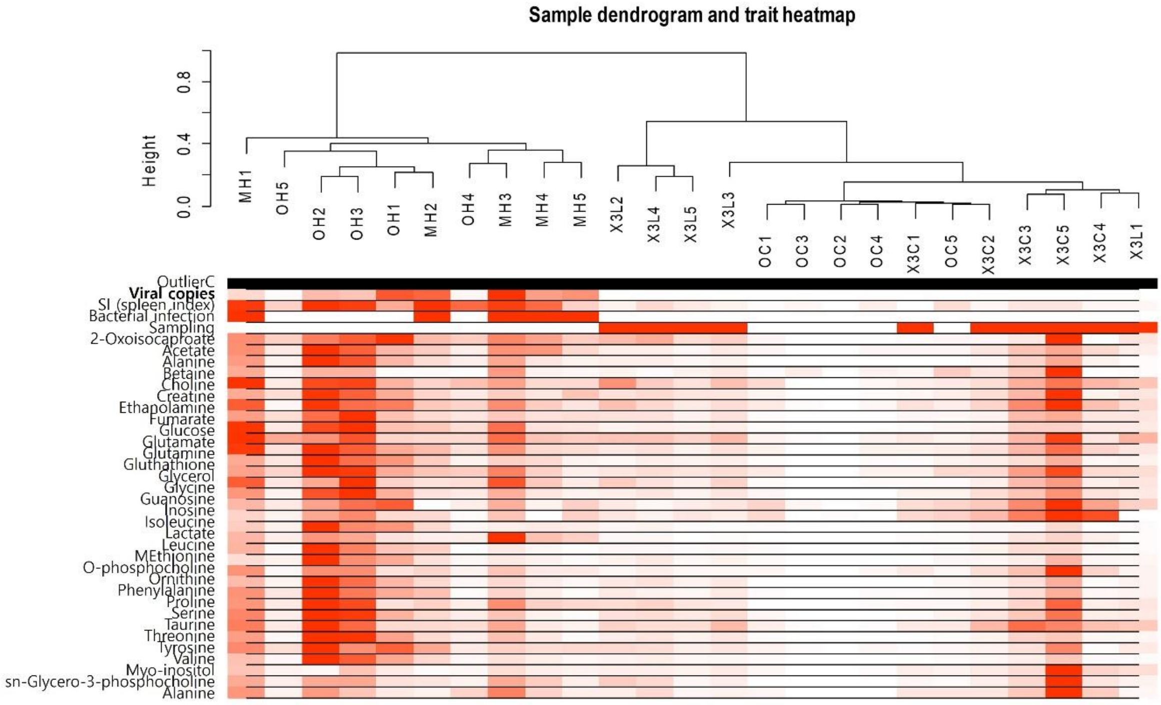

Supplement: Supplementary file 1 [file ijms-21-01707-s001.zip › ijms-690927 supplementary for publish/Figure S1..pdf]
