# Supplementary material for: Co-Expression Network Analysis of Spleen Transcriptome in Rock Bream (Oplegnathus fasciatus) Naturally Infected with Rock Bream Iridovirus (RBIV)
Source: Int J Mol Sci. 2020 Mar 2;21(5):1707. doi: 10.3390/ijms21051707 (PMC7084886; doi:10.3390/ijms21051707)

Figure S2. (A) OPLS-DA score plot of spleen spectra from rock bream ($R^2X = 0.808$, $R^2Y = 0.68$, $Q^2 = 0.274$). (B) VIP scores of contributed metabolites in PLSDA model (top 15 metabolites). Colored boxes indicate relative concentrations of corresponding metabolites in each group.

(A)

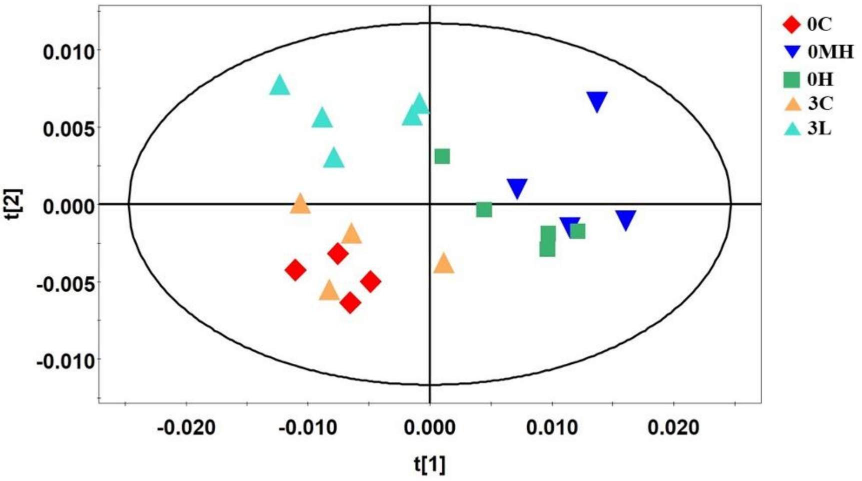

(B)

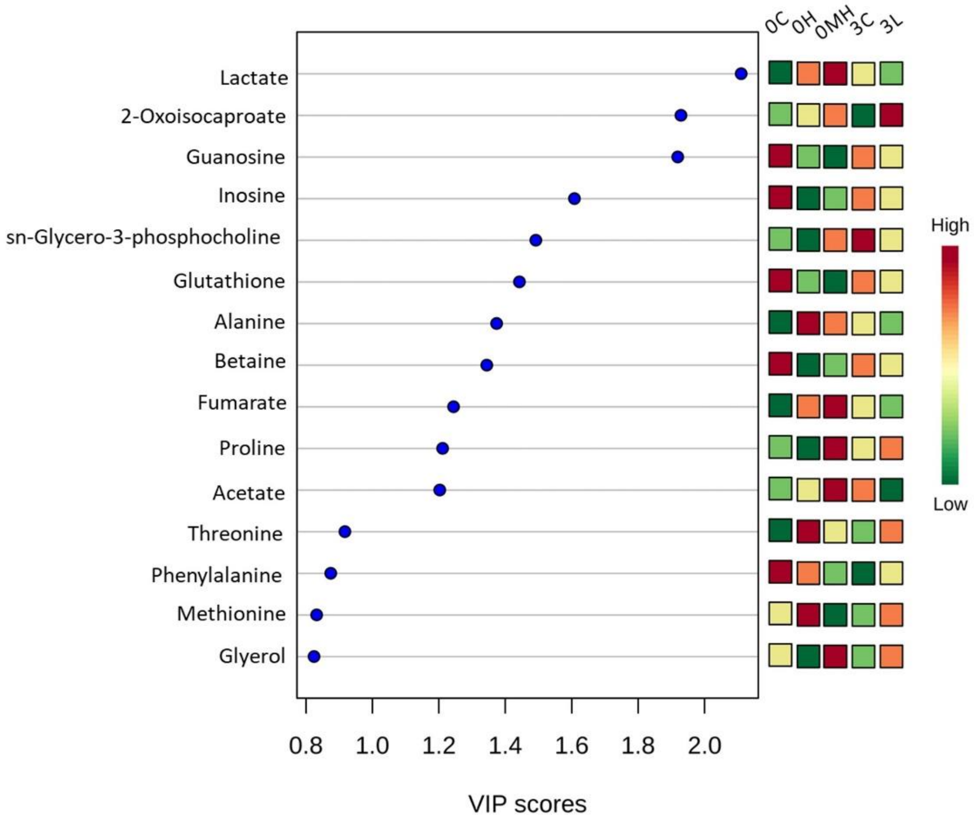

Supplement: Supplementary file 1 [file ijms-21-01707-s001.zip › ijms-690927 supplementary for publish/Figure S2..pdf]
