# Supplementary material for: Co-Expression Network Analysis of Spleen Transcriptome in Rock Bream (Oplegnathus fasciatus) Naturally Infected with Rock Bream Iridovirus (RBIV)
Source: Int J Mol Sci. 2020 Mar 2;21(5):1707. doi: 10.3390/ijms21051707 (PMC7084886; doi:10.3390/ijms21051707)
Supplement: Supplementary file 1 [file ijms-21-01707-s001.zip › ijms-690927 supplementary for publish/Figure S3..pdf]

Figure S3. Relative concentrations of metabolites analyzed with NMR. Red dot and line represent the mean and SD value, respectively.

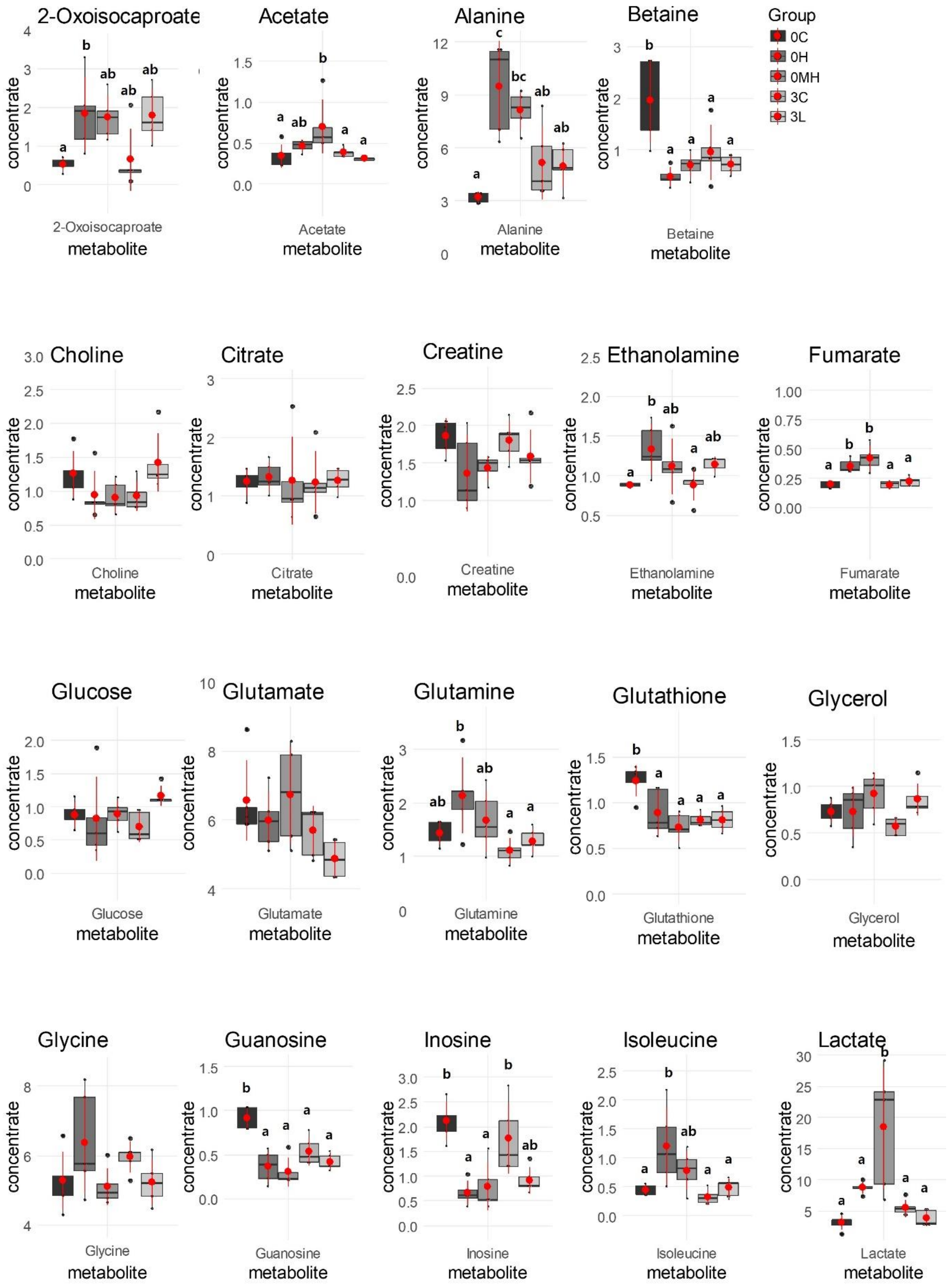

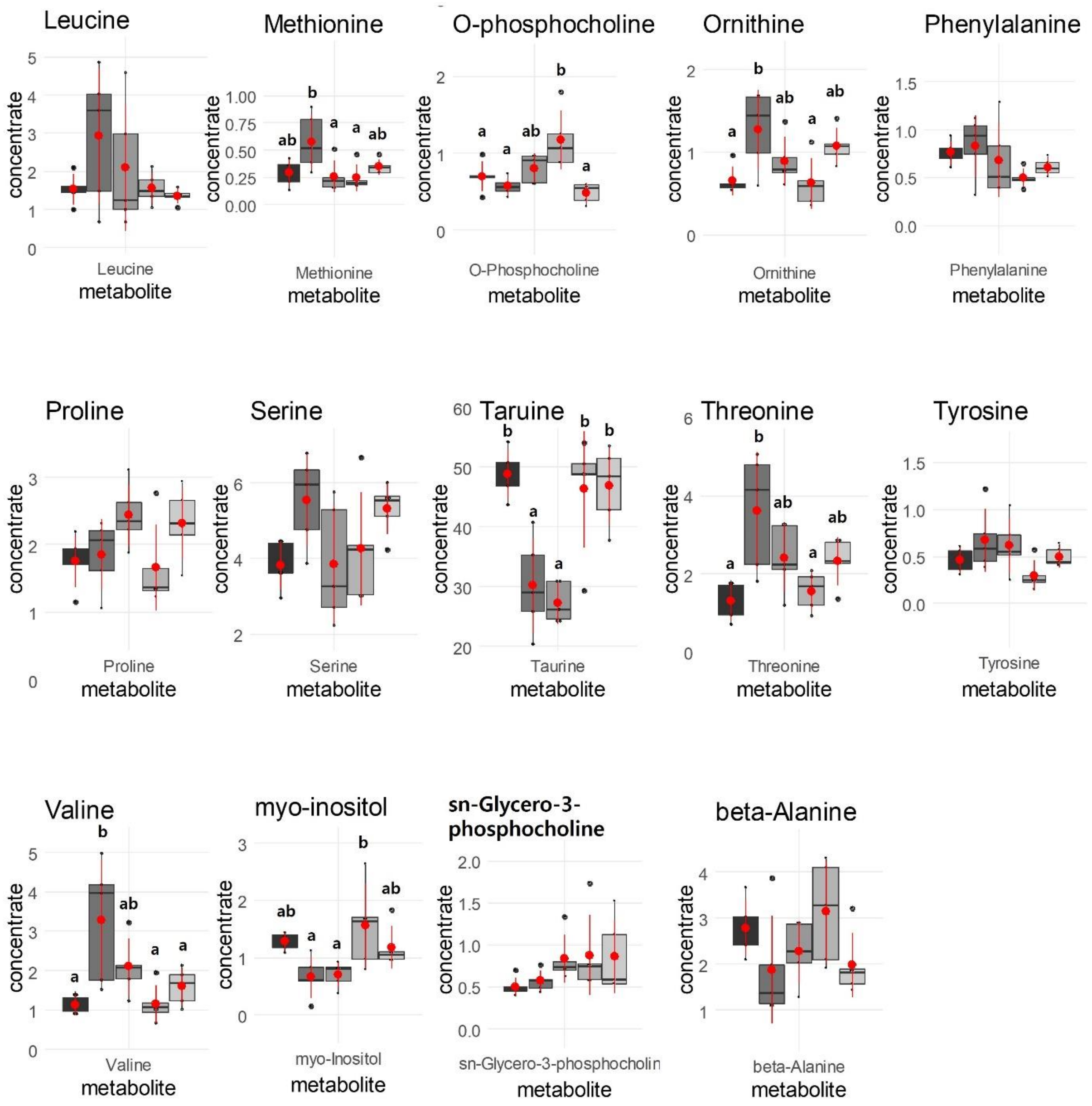

Leucine
concentrate
Leucine
metabolite
Methionine
b
ab
a
a
ab
concentrate
Methionine
metabolite
O-phosphocholine
b
a
a
ab
a
concentrate
O-Phosphocholine
metabolite
Ornithine
b
ab
ab
a
a
concentrate
Ornithine
metabolite
Phenylalanine
concentrate
Phenylalanine
metabolite
Proline
concentrate
Proline
metabolite
Serine
concentrate
Serine
metabolite
Taruine
b
b
b
a
a
concentrate
Taurine
metabolite
Threonine
b
ab
ab
a
a
concentrate
Threonine
metabolite
Tyrosine
concentrate
Tyrosine
metabolite
Valine
b
ab
a
a
a
concentrate
Valine
metabolite
myo-inositol
b
ab
ab
a
a
concentrate
myo-Inositol
metabolite
sn-Glycero-3-phosphocholine
concentrate
sn-Glycero-3-phosphocholin
metabolite
beta-Alanine
concentrate
beta-Alanine
metabolite
