# Supplementary material for: Co-Expression Network Analysis of Spleen Transcriptome in Rock Bream (Oplegnathus fasciatus) Naturally Infected with Rock Bream Iridovirus (RBIV)
Source: Int J Mol Sci. 2020 Mar 2;21(5):1707. doi: 10.3390/ijms21051707 (PMC7084886; doi:10.3390/ijms21051707)

Figure S4. Validation of selected four genes by qPCR. The y-axis represents log_2FC value of gene expression and the x-axis represents DEG groups of 0MH, 0H, 3C, and 3L relative to 0C group. White and gray bars correspond to qPCR and RNA-seq, respectively. Bar and error bar represent mean value and standard deviation (SD), respectively, of five samples in each group.

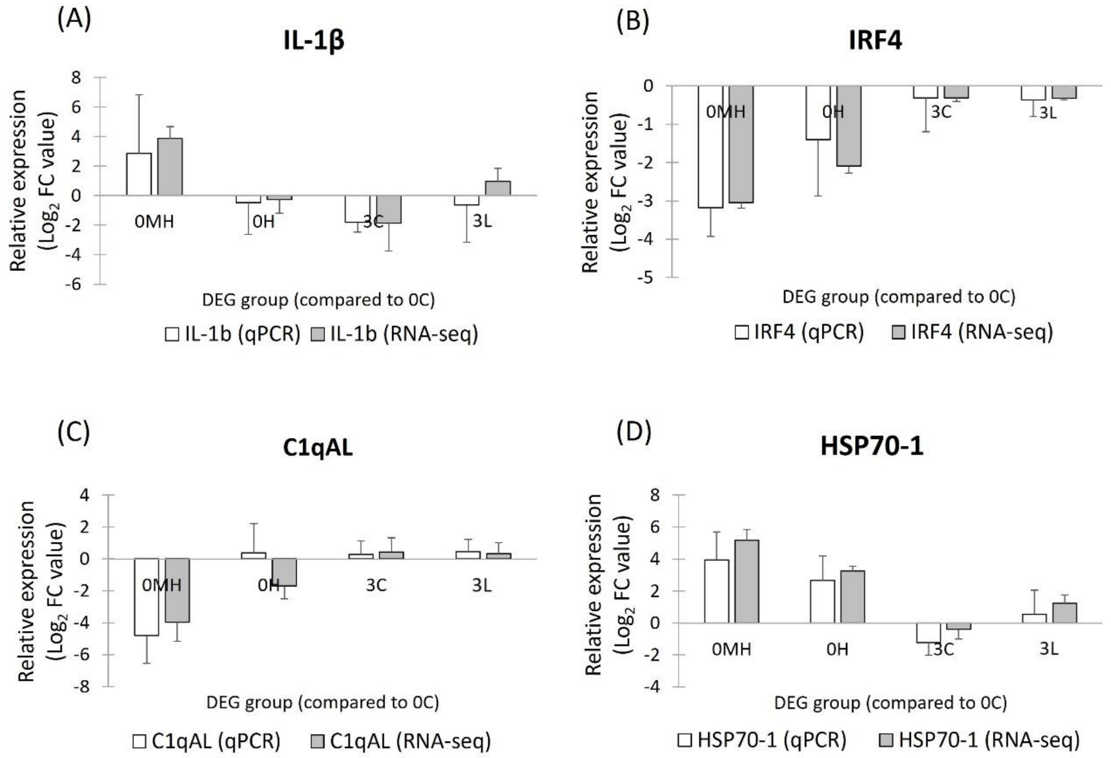

Supplement: Supplementary file 1 [file ijms-21-01707-s001.zip › ijms-690927 supplementary for publish/Figure S4..pdf]
