# Supplementary material for: Co-Expression Network Analysis of Spleen Transcriptome in Rock Bream (Oplegnathus fasciatus) Naturally Infected with Rock Bream Iridovirus (RBIV)
Source: Int J Mol Sci. 2020 Mar 2;21(5):1707. doi: 10.3390/ijms21051707 (PMC7084886; doi:10.3390/ijms21051707)

Figure S5. The amplification and standard curve of MCP gene of RBIV by qPCR. (A) Amplification of 10-fold dilution of plasmid DNA ranging from 10^8 to 10^1 copies/ul, (B) Standard curve generated by plotting Ct values versus 10-fold dilutions of plasmid DNA from 10^8 to 10^1 copies/ul. 1, 1 x 10^1 copies/ul; 2, 1 x 10^2 copies/ul; 3, 1 x 10^3 copies/ul; 4, 1 x 10^4 copies/ul; 5, 1 x 10^5 copies/ul; 6, 1 x 10^6 copies/ul; 7, 1 x 10^7 copies/ul; 8, 1 x 10^8 copies/ul; NC, no-template DNA

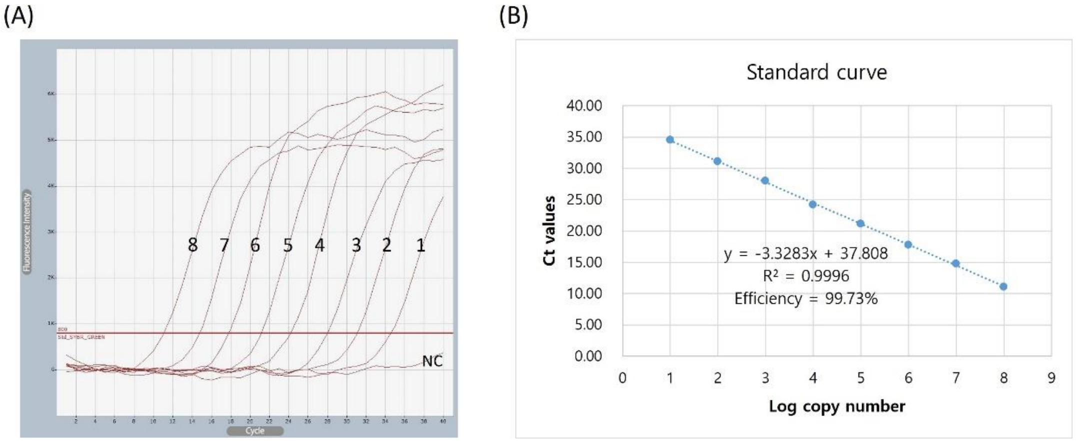

Supplement: Supplementary file 1 [file ijms-21-01707-s001.zip › ijms-690927 supplementary for publish/Figure S5..pdf]
