# Supplementary material for: Co-Expression Network Analysis of Spleen Transcriptome in Rock Bream (Oplegnathus fasciatus) Naturally Infected with Rock Bream Iridovirus (RBIV)
Source: Int J Mol Sci. 2020 Mar 2;21(5):1707. doi: 10.3390/ijms21051707 (PMC7084886; doi:10.3390/ijms21051707)
Supplement: Supplementary file 1 [file ijms-21-01707-s001.zip › ijms-690927 supplementary for publish/Supplementary Methods and Results-Revised2.docx]

**Supplementary Methods**

*RBIV Detection and Quantification of Viral Copy Number in the Spleen*

For RBIV detection, nested PCR was performed with primer sets of M1F/M1R and M2F/M2R (Table S10) in a total volume of 20 µl with 1 µL of each primer (10 pM), 2 µL of genomic DNA (2 µL of 1st-step PCR product in 2nd-step) from spleen, and DEPC water using a MyCycler Thermal Cycler system (BIO-RAD, CA, USA). PCR conditions were as follows: pre-denaturation at 94 °C for 3min, 30 cycles of 94°C for 30 s, annealing and extension at 72 °C for 30 s, followed by post-extension for 7 min at 72 °C.

We also conducted quantitative PCR to confirm viral loads in samples. As a standard, 141 bp amplicon obtained by using a primer set of qM1F and qM1R (Table S10) was inserted into T-easy vector (Promega, USA). This plasmid was then transformed into *Escherichia coli* DH5α. After incubation in LB broth (Difco) at 37°C, recombinant plasmid was isolated using GeneAll Plasmid SV DNA purification kit (GeneAll, Korea). Serial 10-fold dilutions of this plasmid ranging from 10^8^ to 10^1^ copies/µl were used to generate standard curve for RBIV quantification (Table S5). To determine degree of RBIV infection in rock bream, genomic DNAs were isolated from spleen tissues using an AccuPrep Genomic DNA extraction kit (Bioneer, Korea) according to the manufacturer’s instructions. qPCRs were performed using primers qM1F and qM1R in a 20 µl reaction mixture containing 10 µl of qPCR green 2X mastermix kit (m.biotech, Korea), 2 µl of DNA template, 1 µl of each primer (10 pM), and 6 µl of DEPC water using an Exicycler 96 Real-Time Quantitative Thermal Block (Bioneer, Korea). qPCR conditions were as follows: 95 °C for 10 min, followed by 40 cycles of 95 °C for 10 s, 60 °C for 15 s, and 72 °C for 20 s. Results are expressed as viral copy number/mg of spleen.

*Experimental validation with qRT-PCR*

To validate sequences generated from Hiseq2500, several immune-related genes were chosen (Table S10). qRT-PCR was performed using an Exicycler3^TM^ 96 Real-Time Quantitative Thermal Block (Bioneer, Korea). First-strand cDNA was synthesized from 1 µg of total RNA in each sample using a PrimeScript^TM^ RT reagent kit (TAKARA Bio, Japan) according to the manufacturer’s instructions. Quantitative real-time PCR assay was performed as described in section 4.7.

*Library construction and full-length transcript sequencing (Iso-seq)*

Sequencing libraries were prepared according to Iso-Seq™ protocol (Pacific Biosciences, USA). Briefly, cDNA was synthesized according to the protocol of SMART™ cDNA Library Construction Kit (Clontech, USA). Libraries of three different sizes (1–2 kb, 2-3 kb, and 3-6 kb) were selected using a BluePippin™ Size-Selection System (Sage Science, USA), purified, end repaired, and ligated with blunt end SMRTbell adapters. Subsequently, libraries were sequenced on 9 SMRT cells using a PacBio RS II system (Pacific Biosciences, Palo Alto, CA, USA).

*Clustering and CDSs prediction for Iso-Seq results*

Data processing and clustering of raw data from 9 SMRT cells were performed using SMRT® Analysis Software v2.3.0. Briefly, reads of inserts (ROI, minimum read quality of 75) were classified into full-length and non-full-length reads using Iso-Seq classify tool as the first step. For isoform-level clustering, all full-length reads were clustered and assembled into consensus sequences using iterative isoform-clustering algorithm through Iterative Clustering for Error correction (ICE) algorithm. Unpolished consensus and non-full-length reads were then polished using Quiver algorithm. Consensus sequences were clustered into high- and low- quality consensus isoforms based on clustering results of each library using CD-HIT (sequence identity ≥ 0.99) [95]. Coding sequences (CDSs) were predicted with TransDecoder (http://transdecoder.github.io) [96].

*NMR experiment and data analysis*

The spleen (~10 mg) was transferred to an NMR nano tube and 25 µL of D_2_O containing 2 mM TSP-d_4_ was added. Samples were measured using high resolution magic angle spinning nuclear magnetic resonance (HR-MAS NMR) spectroscopy. All spectra were acquired with 600.167 MHz Agilent NMR spectrometer equipped with a 4-mm gHX NanoProbe (Agilent technologies, USA) at a spinning rate of 2,000 Hz. CPMG (Carr-Purcell-Meiboom-Gill) pulse sequence was used to suppress signals from water and high molecular mass compounds. The acquisition time was 1.703 ms and the relaxation delay was 1 s. A total of 128 transients were collected. All spectra were processed and assigned using Chenomx NMR Suite 7.1 professional with the Chenomx 600 MHz library database (Chenomx Inc., Canada). Spectra were binned for multivariate statistical analysis. The binning size was 0.001 ppm. Normalization was performed for all spectral binning data to the total area of the NMR spectrum, and the relative concentration normalized by the sum of the absolute concentrations of the measured metabolite was used in order to reduce the variation between samples. Statistical analyses on each metabolite were conducted using one-way analysis of variance (ANOVA) with a Tukey’s post hoc test for multiple comparisons. All data were analyzed with SPSS 16.0 software (SPSS Inc.). Differences were considered significant at p < 0.05.

*Integration of Transcriptome and Metabolite Data*

To understand biologically functional differences through integrating expressed genes and metabolites, web-based Integrated Molecular Pathway-Level Analysis (IMPaLA) (http://impala.molgen.mpg.de) [97] was performed. Since genes in each module and metabolites had different values for each group, Wilcoxon pathway enrichment analysis (WEA) was performed by inputting gene symbols of DEGs and corresponding log_2_FC value for eigengenes in each module as well as metabolite names and logFC value of relative concentration for metabolite. All expression and concentration values were calculated and compared to those of the 0C group. Pathways in KEGG database among results obtained from 11 databases were chosen. It was analyzed if at least one gene and metabolite were jointed to derive integration result of genes and metabolites. Joint p-value was less than 0.05.

**Supplementary Results**

*Transcriptome overview*

We obtained 4,395,653 raw reads from 9 SMRTcells on PacBio RS II chemistry, with a total 13,174,676,754 bases and an average length of 2,997 bases per read (Tables S7A,7B). Finally, Iso-seq produced 68,211 unigenes with a total of 207,448,683 bases and an average length of 3,041 bases per read. Of these, 60,060 genes (88.05%) were annotated with top hits based on sequence homology search using BLASTx (Table S7C). From Illumina HiSeq 2500, an average of 5.326 Gb per sample was generated. After trimming adapter sequences and low-quality reads, an average of 84.75% from 5.30 Gb of clean reads per sample were mapped onto unigenes and approximately 78.76% were aligned with paired reads among mapped reads (Table S8).

*Validation by qPCR*

We compared expression levels of four immune-related genes (IRF4, IL-1β, C1qAL, and HSP70-1) among genes derived from *Oplegnathus fasciatus* that were annotated with BLASTx. Figure S4 demonstrates that RNA-seq and qPCR data are very similar, indicating that expression profiles are reliable.

*NMR-based metabolomics and integrated analysis with transcriptome and metabolome*

Normalized spectra of spleen were subjected to orthogonal partial least-squares discriminate analysis (OPLS-DA) to differentiate between groups (Figure S2A). Metabolic profiles of the spleen were classified according to the degree of infection. Groups 0C and 3C were clustered in the negative area of t [1] while groups 0H and 0MH were clustered in the positive area of t [1]. Spots of 3L group were located in the negative area of t [1] with two control groups, but distinguished from control groups by clustering in the positive area of t [2]. This pattern clearly indicated different metabolite changes between heavy infection groups and control groups. The recovery group also differed from heavy infection groups and control groups. Eleven metabolites recorded good scores (> 1.0) of variable influence on projection (VIP). Thus, they were considered as significant contributors to differences in the PLSDA model (Figure S2B).

To investigate significant differences in metabolites of each group, relative concentrations of 33 metabolites were determined (Figure S3). Lactate and taurine accounted for the highest proportions in all groups. Significant differences included betaine, glutathione, guanosine, inosine, and taurine in 0C group, inosine, O-phosphocholine, *myo*-inositol, and taurine in 3C group, and taurine in 3L group. Alanine, 2-oxoisocaproate, ethanolamine, fumarate, isoleucine, methionine, ornithine, glutamine, threonine, and valine in 0H group and acetate, fumarate, and lactate in 0MH group were also significantly different. By combining these results, 2-oxoisocaproate, alanine, acetate, fumarate, and lactate concentrations were elevated while betaine, glutathione, guanosine, and inosine concentrations were reduced in heavily infected groups than those in other groups, suggesting that these changes of metabolites might contribute to differences between groups.

As a result of over-representation pathway analysis (ORA) of 33 metabolites carried out with IMPaLA, 56 pathways satisfying *p*-value < 0.05 in KEGG database are shown in Table S3. Analyzed metabolites were associated with central carbon metabolism in cancer, protein digestion and absorption, ABC transporters, mineral absorption pathway, and aminoacyl-tRNA biosynthesis (Table S3). All results showed only pathways related to metabolism.

We employed WEA method to reflect each fold change for each group compared to 0C and confirmed differences between groups when genes in each module and metabolites were integrated. Among KEGG pathways analyzed with a joint *p*-value < 0.05, only two pathways with *p*-value < 0.05 in both transcriptome and metabolite pathway results were found: aminoacyl-tRNA biosynthesis and central carbon metabolism in cancer in Turquoise and protein digestion and absorption in Blue (Table S4).
