# Supplementary material for: Co-Expression Network Analysis of Spleen Transcriptome in Rock Bream (Oplegnathus fasciatus) Naturally Infected with Rock Bream Iridovirus (RBIV)
Source: Int J Mol Sci. 2020 Mar 2;21(5):1707. doi: 10.3390/ijms21051707 (PMC7084886; doi:10.3390/ijms21051707)
Supplement: Supplementary file 1 [file ijms-21-01707-s001.zip › ijms-690927 supplementary for publish/Table S1..docx]

**Table S1.** Viral load and spleen index (SI) of rock bream sample used in this study. Statistics analysis was performed with ANOVA for multiple comparisons (SPSS 16.0 software, *p*-value < 0.05).

| **Group** | **Sample** | **Viral copies/mg of spleen** | **Average of vial copies/mg of spleen** | **Spleen weight (mg)** | **Body weight (g)** | **Spleen index** | **Average of spleen index** | **Isolated bacterial species** |
| --- | --- | --- | --- | --- | --- | --- | --- | --- |
| 0C | 1 | 1.17 × 10^2^ | 7.07 ± 4.14 × 10^1 a^ | 21.00 | 19.68 | 1.07 | 0.85 ± 0.14^a^ | ND |
|  | 2 | 9.80 × 10^1^ |  | 16.20 | 17.82 | 0.91 |  | ND |
|  | 3 | 7.88 × 10^1^ |  | 12.00 | 16.41 | 0.73 |  | ND |
|  | 4 | 4.75 × 10^1^ |  | 15.30 | 18.61 | 0.82 |  | ND |
|  | 5 | 1.26 ×10^1^ |  | 14.00 | 19.04 | 0.74 |  | ND |
| 0MH | 1 | 4.33 × 10^8^ | 2.56 ± 1.31 × 10^8 b^ | 94.70 | 43.91 | 2.16 | 2.15 ± 0.56^b^ | VS, VH |
|  | 2 | 3.25 × 10^8^ |  | 54.50 | 22.60 | 2.41 |  | VH |
|  | 3 | 2.34 × 10^8^ |  | 36.00 | 22.39 | 1.61 |  | VS, VH |
|  | 4 | 2.04 × 10^8^ |  | 47.20 | 28.94 | 1.63 |  | VS, VH |
|  | 5 | 4.16 × 10^7^ |  | 91.90 | 31.4 | 2.93 |  | VH |
| 0H | 1 | 3.46 × 10^8^ | 1.30 ± 1.36 × 10^8 ab^ | 45.10 | 27.05 | 1.67 | 2.26 ± 0.79^b^ | ND |
|  | 2 | 1.44 × 10^8^ |  | 115.40 | 37.7 | 3.06 |  | ND |
|  | 3 | 1.29 × 10^8^ |  | 96.70 | 33.23 | 2.91 |  | ND |
|  | 4 | 1.84 × 10^7^ |  | 60.90 | 24.91 | 2.44 |  | ND |
|  | 5 | 1.01 × 10^7^ |  | 23.50 | 19.3 | 1.22 |  | ND |
| 3C | 1 | ND | - ^a^ | 26.10 | 37.96 | 0.69 | 0.86 ± 0.14^a^ | ND |
|  | 2 | ND |  | 34.90 | 43.71 | 0.80 |  | ND |
|  | 3 | ND |  | 46.90 | 46.4 | 1.01 |  | ND |
|  | 4 | ND |  | 37.10 | 45.12 | 0.82 |  | ND |
|  | 5 | ND |  | 37.10 | 37.24 | 1.00 |  | ND |
| 3L | 1 | 1.10 × 10^4^ | 4.15 ± 4.14 × 10^3 a^ | 26.40 | 31.99 | 0.83 | 0.92 ± 0.13^a^ | ND |
|  | 2 | 4.92 × 10^3^ |  | 28.20 | 33.64 | 0.84 |  | ND |
|  | 3 | 2.79 × 10^3^ |  | 30.90 | 27.01 | 1.14 |  | ND |
|  | 4 | 1.47 × 10^3^ |  | 27.30 | 32.28 | 0.85 |  | ND |
|  | 5 | 6.28 × 10^2^ |  | 39.70 | 42.9 | 0.93 |  | ND |

ND, no detection; VS, *Vibrio scophthalmi*; VH, *V. harveyi*
