# Supplementary material for: Co-Expression Network Analysis of Spleen Transcriptome in Rock Bream (Oplegnathus fasciatus) Naturally Infected with Rock Bream Iridovirus (RBIV)
Source: Int J Mol Sci. 2020 Mar 2;21(5):1707. doi: 10.3390/ijms21051707 (PMC7084886; doi:10.3390/ijms21051707)
Supplement: Supplementary file 1 [file ijms-21-01707-s001.zip › ijms-690927 supplementary for publish/Table S10..docx]

**Table S10.** Primers used for virus detection, virus quantification, and NGS validation.

| Genes | Name | Sequences (5’ to 3’) | Size (bp) | Annealing Temperature (**℃)** | Efficiency (%) | Purpose | References |
| --- | --- | --- | --- | --- | --- | --- | --- |
| MCP | qM1F | GGCGACTACCTCATTAATGT | 141 | 60 | 99.73 | qPCR for quantification of RBIV | [98] |
|  | qM1R | CCACCAGGTCGTTAAATGA |  |  |  |  |  |
|  | M1F | GCTGCGCATGCCAATCATCT | 401 |  |  | 1-step PCR for RBIV detection | [99] |
|  | M1R | ATGCGATGGAGACCCACTTG |  |  |  |  |  |
|  | M2F | AATGACACCGACACCTCCTC | 288 |  |  | 2-step PCR for RBIV detection |  |
|  | M2R | TGCGATGGAGACCCACTTGT |  |  |  |  |  |
| β-actin | Rbβ-actin-F | TCATCACCATCGGCAATGAGAGGT | 108 | 58 | 99.70 | qRT-PCR amplification | [91] |
|  | Rbβ-actin-R | TGATGCTGTTGTAGGTGGTCTCGT |  |  |  |  |  |
| IRF4 | RbIRF4-F | ATGGCTTATACGCTAAGCGCCTCT | 221 | 58 | 98.58 | qRT-PCR amplification | [92] |
|  | RbIRF4-R | GTTTGCATGGCTGTTCCTTCTCCA |  |  |  |  |  |
| IL-1b | rbIL1b F | ATCTGGAGACGGTGGACAAC | 142 | 60 | 100.35 | qRT-PCR amplification | [18] |
|  | rbIL1b R | GCTGATGTACCAGTCGCTGA |  |  |  |  |  |
| C1qAL | RbC1qALF | TGAGCAACAAGCTGGGATTCTGTG | 124 | 58 | 102.46 | qRT-PCR amplification | [93] |
|  | RbC1qAL R | AAGGACTCGAGCCAAACCTTCTGT |  |  |  |  |  |
| HSP70-1 | RbHSP70-1F | ACAAAAGCACCGGCAAAGAG | 144 | 58 | 99.25 | qRT-PCR amplification | In this study |
|  | RbHSP70-1R | TCTTGGCAGCGATTTTCTCC |  |  |  |  |  |

* Efficiency (%) = (10^−1/slope^ – 1) X 100
