# Supplementary material for: Co-Expression Network Analysis of Spleen Transcriptome in Rock Bream (Oplegnathus fasciatus) Naturally Infected with Rock Bream Iridovirus (RBIV)
Source: Int J Mol Sci. 2020 Mar 2;21(5):1707. doi: 10.3390/ijms21051707 (PMC7084886; doi:10.3390/ijms21051707)
Supplement: Supplementary file 1 [file ijms-21-01707-s001.zip › ijms-690927 supplementary for publish/Table S3..docx]

**Table S3.** Over-represented KEGG pathways (*p*-value < 0.05) of 33 metabolites performed by IMPaLA.

| KEGG pathway name | All counts in pathway | Metabolite count | (%) | Overlapping metabolites | *p*-value |
| --- | --- | --- | --- | --- | --- |
| Central carbon metabolism in cancer | 37 | 16 | 43.24 | Citrate, Glutamine, Alanine, Methionine, Serine, Lactate, Glucose, Glycine, Glutamate, Phenylalanine, Isoleucine, Proline, Tyrosine, Valine, Leucine, Fumarate | 1.37E-26 |
| Protein digestion and absorption | 47 | 15 | 31.91 | Glutamine, Serine, Proline, Methionine, Threonine, Acetate, Valine, Glycine, Isoleucine, Phenylalanine, Glutamate, Tyrosine, Beta-alanine, Alanine, Leucine | 1.88E-22 |
| ABC transporters | 129 | 19 | 14.73 | Glutamine, Serine, Glycerol, Proline, Taurine, Threonine, Valine, Ornithine, Glycine, Isoleucine, Phenylalanine, Glutamate, Choline, Betaine, Myo-inositol, Alanine, Glutathione, Glucose, Leucine | 1.01E-21 |
| Mineral absorption | 29 | 12 | 41.38 | Glutamine, Serine, Proline, Methionine, Threonine, Valine, Glucose, Glycine, Isoleucine, Phenylalanine, Alanine, Leucine | 1.47E-19 |
| Aminoacyl-tRNA biosynthesis | 52 | 13 | 25.00 | Glutamine, Serine, Proline, Methionine, Threonine, Valine, Glycine, Isoleucine, Phenylalanine, Glutamate, Tyrosine, Alanine, Leucine | 7.80E-18 |
| Valine, leucine and isoleucine biosynthesis | 23 | 5 | 21.74 | Leucine, Valine, Isoleucine, 2-Oxoisocaproate, Threonine | 5.14E-07 |
| Glycine, serine and threonine metabolism | 50 | 6 | 12.00 | Choline, Serine, Threonine, Glycine, Creatine, Betaine | 1.34E-06 |
| Alanine, aspartate and glutamate metabolism | 28 | 5 | 17.86 | Citrate, Glutamine, Alanine, Glutamate, Fumarate | 1.46E-06 |
| Glyoxylate and dicarboxylate metabolism | 62 | 6 | 9.68 | Citrate, Serine, Acetate, Glycine, Glutamate, Glutamine | 4.87E-06 |
| Taurine and hypotaurine metabolism | 22 | 4 | 18.18 | Acetate, Alanine, Taurine, Glutamate | 1.70E-05 |
| Arginine biosynthesis | 23 | 4 | 17.39 | Glutamine, Ornithine, Glutamate, Fumarate | 2.05E-05 |
| Glucagon signaling pathway | 26 | 4 | 15.38 | Citrate, Lactate, Glucose, Fumarate | 3.41E-05 |
| Choline metabolism in cancer | 11 | 3 | 27.27 | Choline, O-Phosphocholine, sn-Glycero-3-phosphocholine | 5.98E-05 |
| Glutathione metabolism | 38 | 4 | 10.53 | Ornithine, Glutathione, Glycine, Glutamate | 0.000158 |
| Valine, leucine and isoleucine degradation | 42 | 4 | 9.52 | Leucine, Valine, Isoleucine, 2-Oxoisocaproate | 0.000235 |
| Retrograde endocannabinoid signaling | 19 | 3 | 15.79 | Glycerol, Glutamate, Ethanolamine | 0.000337 |
| FoxO signaling pathway | 5 | 2 | 40.00 | Glucose, Glutamate | 0.000531 |
| Glycerophospholipid metabolism | 52 | 4 | 7.69 | Choline, O-Phosphocholine, sn-Glycero-3-phosphocholine, Ethanolamine | 0.000539 |
| Cysteine and methionine metabolism | 61 | 4 | 6.56 | Serine, Alanine, Methionine, Glutathione | 0.00099 |
| Cocaine addiction | 8 | 2 | 25.00 | Tyrosine, Glutamate | 0.00147 |
| Glutamatergic synapse | 8 | 2 | 25.00 | Glutamine, Glutamate | 0.00147 |
| Pyruvate metabolism | 31 | 3 | 9.68 | Acetate, Lactate, Fumarate | 0.00147 |
| Glycolysis / Gluconeogenesis | 31 | 3 | 9.68 | Acetate, Lactate, Glucose | 0.00147 |
| Sulfur metabolism | 33 | 3 | 9.09 | Acetate, Serine, Taurine | 0.00177 |
| Taste transduction | 33 | 3 | 9.09 | Citrate, Glucose, Glutamate | 0.00177 |
| GABAergic synapse | 9 | 2 | 22.22 | Glutamine, Glutamate | 0.00188 |
| Alcoholism | 10 | 2 | 20.00 | Tyrosine, Glutamate | 0.00234 |
| Amphetamine addiction | 10 | 2 | 20.00 | Tyrosine, Glutamate | 0.00234 |
| Arginine and proline metabolism | 78 | 4 | 5.13 | Ornithine, Proline, Glutamate, Creatine | 0.00248 |
| Prolactin signaling pathway | 11 | 2 | 18.18 | Glucose, Tyrosine | 0.00284 |
| D-Glutamine and D-glutamate metabolism | 12 | 2 | 16.67 | Glutamine, Glutamate | 0.00339 |
| Synaptic vesicle cycle | 12 | 2 | 16.67 | Glycine, Glutamate | 0.00339 |
| Cholinergic synapse | 12 | 2 | 16.67 | Choline, Acetate | 0.00339 |
| Galactose metabolism | 46 | 3 | 6.52 | Glycerol, Glucose, Myo-inositol | 0.00461 |
| Purine metabolism | 95 | 4 | 4.21 | Glutamine, Guanosine, Inosine, Glycine | 0.00506 |
| Propanoate metabolism | 48 | 3 | 6.25 | Acetate, Lactate, Beta-alanine | 0.0052 |
| HIF-1 signaling pathway | 15 | 2 | 13.33 | Lactate, Glucose | 0.00533 |
| Proximal tubule bicarbonate reclamation | 17 | 2 | 11.76 | Glutamine, Glutamate | 0.00683 |
| Nitrogen metabolism | 19 | 2 | 10.53 | Glutamine, Glutamate | 0.00851 |
| Citrate cycle (TCA cycle) | 20 | 2 | 10.00 | Citrate, Fumarate | 0.00942 |
| Non-alcoholic fatty liver disease (NAFLD) | 2 | 1 | 50.00 | Glucose | 0.0149 |
| Neuroactive ligand-receptor interaction | 130 | 4 | 3.08 | Beta-alanine, Glycine, Taurine, Glutamate | 0.015 |
| Phenylalanine metabolism | 72 | 3 | 4.17 | Tyrosine, Phenylalanine, Fumarate | 0.0159 |
| Carbohydrate digestion and absorption | 27 | 2 | 7.41 | Acetate, Glucose | 0.0168 |
| Pantothenate and CoA biosynthesis | 28 | 2 | 7.14 | Beta-alanine, Valine | 0.018 |
| Thiamine metabolism | 31 | 2 | 6.45 | Glycine, Tyrosine | 0.0219 |
| Ferroptosis | 31 | 2 | 6.45 | Glutathione, Glutamate | 0.0219 |
| Huntington disease | 3 | 1 | 33.33 | Glutamate | 0.0222 |
| mTOR signaling pathway | 3 | 1 | 33.33 | Leucine | 0.0222 |
| Renal cell carcinoma | 3 | 1 | 33.33 | Fumarate | 0.0222 |
| Phenylalanine, tyrosine and tryptophan biosynthesis | 35 | 2 | 5.71 | Tyrosine, Phenylalanine | 0.0275 |
| Insulin signaling pathway | 4 | 1 | 25.00 | Glucose | 0.0295 |
| Butanoate metabolism | 42 | 2 | 4.76 | Glutamate, Fumarate | 0.0385 |
| Type II diabetes mellitus | 6 | 1 | 16.67 | Glucose | 0.0439 |
| Melanogenesis | 6 | 1 | 16.67 | Tyrosine | 0.0439 |
| Primary bile acid biosynthesis | 47 | 2 | 4.26 | Glycine, Taurine | 0.0473 |
