# Supplementary material for: Co-Expression Network Analysis of Spleen Transcriptome in Rock Bream (Oplegnathus fasciatus) Naturally Infected with Rock Bream Iridovirus (RBIV)
Source: Int J Mol Sci. 2020 Mar 2;21(5):1707. doi: 10.3390/ijms21051707 (PMC7084886; doi:10.3390/ijms21051707)
Supplement: Supplementary file 1 [file ijms-21-01707-s001.zip › ijms-690927 supplementary for publish/Table S4..docx]

**Table S4.** KEGG pathways integrated of transcriptome (*p*-value < 0.05) and metabolite (*p* < 0.05) data through Wilcoxon pathway enrichment analysis (joint *p*-value < 0.05) in IMPaLA.

|  | Pathway name | Transcripts | | | Metabolites | | | joint *p*-value | | | |
| --- | --- | --- | --- | --- | --- | --- | --- | --- | --- | --- | --- |
|  |  | Overlapped counts | All counts in pathway | *p*-value | Overlapped counts | All counts in pathway | *p*-value | 0Cvs 0MH | 0Cvs 0H | 0Cvs 3C | 0Cvs 3L |
| Turquoise | Aminoacyl-tRNA biosynthesis | 31 | 66 | 9.31E-10 | 13 | 52 | 0.0171 | 4.12E-10 | 3.24E-11 | - | - |
|  | Central carbon metabolism in cancer | 14 | 65 | 0.000122 | 16 | 37 | 0.00763 | 1.39E-05 | 1.60E-06 | - | - |
| Blue | Protein digestion and absorption | 6 | 90 | 0.0313 | 15 | 47 | 0.00427 | 0.00479 | 0.00132 | - | - |
