# Supplementary material for: Co-Expression Network Analysis of Spleen Transcriptome in Rock Bream (Oplegnathus fasciatus) Naturally Infected with Rock Bream Iridovirus (RBIV)
Source: Int J Mol Sci. 2020 Mar 2;21(5):1707. doi: 10.3390/ijms21051707 (PMC7084886; doi:10.3390/ijms21051707)
Supplement: Supplementary file 1 [file ijms-21-01707-s001.zip › ijms-690927 supplementary for publish/Table S7..docx]

Table S7. Primers used for gene expression in blood cells of rock bream in this study.

| **Gene** | | **Description (Accession No.)** |  | **Sequence (5’→3’)** | **Size (bp)** | **Annealing Temperature (℃)** | **Efficiency (%)** | **Unigene** | **References** |
| --- | --- | --- | --- | --- | --- | --- | --- | --- | --- |
| Hub gene | PRPF19 | Pre-mRNA-processing factor 19 (Q9UMS4) | F | TCGCCAAGAATTGTCACACG | 85 | 58 | 98.23 | RockBream_ISO000111 | In this study |
|  |  |  | R | TGCAGCAGTAACCTCTTTGG |  |  |  |  |  |
| Apoptosis | CASP3 | Caspase-3 | F | AGCAGTGTGGAAGAGCAAAC | 143 | 59 | 99.04 | RockBream_ISO018528  RockBream_ISO045470 | In this study |
|  |  | (Q60431) | R | TGCAACATCTCACACAAGGC |  |  |  |  |  |
| Pattern recognition receptor (PRR) | TLR9 | Toll-like receptor 9 (Q9NR96) | F | TGGAGAACTCGAATCACAGGAG | 72 | 59 | 101.14 | RockBream_ISO055388 | In this study |
|  |  |  | R | TGCATGAAAGCCCAGGAATC |  |  |  |  |  |
| Immune-related gene | CTLA4-L | Cytotoxic T-lymphocyte protein 4-like  (NR 734643188) | F | TGACAGTGTCTGGACTGCAAG | 110 | 59 | 98.44 | RockBream_ISO065023 | In this study |
|  |  |  | R | ATGAATGAGTGTGCCGTTGC |  |  |  |  |  |
| Antigen presentation | MHCI (H2L) | H-2 class I histocompatibility antigen, L-D alpha chain (P01897) | F | AGATTACTGGGAAAAAGGCACA | 86 | 60 | 101.14 | RockBream_ISO051992 | [100] |
|  |  |  | R | TCATTCGTTTCATCAGGATGTC |  |  |  |  |  |
|  | MHC2α | MHC class II antigen alpha chain (Q30631) | F | ACGCAGACTTCAGCAACAAC | 94 | 59 | 99.46 | RockBream_ISO002795 | In this study |
|  |  |  | R | GCCACAGCTGTTTGATAAGCTC |  |  |  |  |  |
| Platelet activation | ITGA2B | Integrin alpha 2B (P08514) | F | TGTGTGTGTTTGGGGATTCG | 86 | 58 | 97.43 | RockBream_ISO023674  RockBream_ISO038373 | In this study |
|  |  |  | R | TCGGTGTTGTTGTCAGTGTC |  |  |  |  |  |
|  | GP5 | Platelet glycoprotein V (O08770) | F | TGTATGCCACTAACCTCACCAC | 147 | 60 | 100.29 | RockBream_ISO035817 |  |
|  |  |  | R | ACGAGAGCTTCTGAAGGTTGG |  |  |  |  |  |

* Efficiency (%) = (10^−1/slope^ – 1) X 100
