# Supplementary material for: Co-Expression Network Analysis of Spleen Transcriptome in Rock Bream (Oplegnathus fasciatus) Naturally Infected with Rock Bream Iridovirus (RBIV)
Source: Int J Mol Sci. 2020 Mar 2;21(5):1707. doi: 10.3390/ijms21051707 (PMC7084886; doi:10.3390/ijms21051707)
Supplement: Supplementary file 1 [file ijms-21-01707-s001.zip › ijms-690927 supplementary for publish/Table S8..docx]

**Table S8.** Statistics of transcript sequencing obtained from PacBio P6C4. (A) Raw data and (B) subreads after clustering in each library, and (C) statistics of annotation of unigenes generated by Iso-seq.

| 1. **Raw data** | | | | |
| --- | --- | --- | --- | --- |
| **Cell Name** | **Subread Bases** | **Subreads** | | **Average Subread length** |
| 1-2kb_1 | 1,455,078,316 | 587,073 | | 2,479 |
| 1-2kb_2 | 1,463,138,306 | 594,859 | | 2,460 |
| 1-2kb_3 | 1,348,833,129 | 557,847 | | 2,418 |
| 2-3kb_1 | 1,638,599,355 | 517,536 | | 3,166 |
| 2-3kb_2 | 1,558,748,622 | 521,204 | | 2,991 |
| 2-3kb_3 | 1,552,041,194 | 487,069 | | 3,186 |
| 3-6kb_1 | 1,349,726,990 | 361,735 | | 3,731 |
| 3-6kb_2 | 1,408,078,020 | 388,146 | | 3,628 |
| 3-6kb_3 | 1,400,432,822 | 380,184 | | 3,684 |
| total | 13,174,676,754 | 4,395,653 | | 2,997 |
| 1. **Subreads after clustering from each library** | | | | |
| **Library** | **Number of consensus isoforms** | | **Average consensus isoforms read length** | |
| 1-2kb high quality | 19,633 | | 2,065 | |
| 1-2kb low quality | 6,411 | | 2,349 | |
| 2-3kb high quality | 21,453 | | 3,007 | |
| 2-3kb low quality | 13,308 | | 3,274 | |
| 3-6kb high quality | 11,970 | | 3,604 | |
| 3-6kb low quality | 11,424 | | 4,151 | |
| **(C) Annotation of unigenes** | | | | |
| **Database** | **No. of genes (%)** | | | |
| Swissprot | 53,889 (79.00) | | | |
| NCBI nr | 59,949 (87.89) | | | |
| InterProScan | 45,829 (67.19) | | | |
| GO | 34,214 (50.16) | | | |
| KEGG | 15,389 (22.56) | | | |
| Total | 60,060 (88.05) | | | |
