# Supplementary material for: Co-Expression Network Analysis of Spleen Transcriptome in Rock Bream (Oplegnathus fasciatus) Naturally Infected with Rock Bream Iridovirus (RBIV)
Source: Int J Mol Sci. 2020 Mar 2;21(5):1707. doi: 10.3390/ijms21051707 (PMC7084886; doi:10.3390/ijms21051707)
Supplement: Supplementary file 1 [file ijms-21-01707-s001.zip › ijms-690927 supplementary for publish/Table S9..docx]

**Table S9.** Statistics of transcript sequencing obtained from Illumina Hi-Seq2500.

|  | **Raw reads** | | | **Clean reads** | | | **Mapped onto Iso-seq reference unigene** | |
| --- | --- | --- | --- | --- | --- | --- | --- | --- |
| **SampleID** | **Total Reads** | **Total**  **Bases (Gb)** | **GC Rate** | **Total Reads** | **Total Bases (Gb)** | **GC Rate** | **Mapped Reads (%)** | **Paired Reads (%)** |
| 3C1 | 59,187,336 | 5.98 Gb | 49.57% | 59,093,116 | 5.96 Gb | 49.61% | 48,351,283 (81.82%) | 44,770,016 (75.76%) |
| 3C2 | 46,769,012 | 4.72 Gb | 50.06% | 46,689,404 | 4.71 Gb | 50.10% | 39,473,374 (84.54%) | 36,469,280 (78.11%) |
| 3C3 | 58,872,800 | 5.95 Gb | 49.44% | 58,776,636 | 5.92 Gb | 49.48% | 49,242,022 (83.78%) | 45,604,378 (77.59%) |
| 3C4 | 54,416,854 | 5.50 Gb | 48.98% | 54,318,670 | 5.47 Gb | 49.01% | 46,913,237 (86.37%) | 43,479,584 (80.05%) |
| 3C5 | 57,807,194 | 5.84 Gb | 49.77% | 57,704,840 | 5.81 Gb | 49.80% | 48,249,931 (83.62%) | 44,864,774 (77.75%) |
| 3R1 | 54,087,814 | 5.46 Gb | 49.43% | 54,000,120 | 5.44 Gb | 49.45% | 46,000,584 (85.19%) | 42,835,754 (79.33%) |
| 3R2 | 53,641,772 | 5.42 Gb | 49.48% | 53,547,274 | 5.39 Gb | 49.51% | 44,266,301 (82.67%) | 41,110,166 (76.77%) |
| 3R3 | 52,344,968 | 5.29 Gb | 49.83% | 52,242,990 | 5.26 Gb | 49.86% | 43,122,213 (82.54%) | 39,687,072 (75.97%) |
| 3R4 | 51,354,716 | 5.19 Gb | 49.84% | 51,262,912 | 5.16 Gb | 49.87% | 43,543,883 (84.94%) | 40,070,438 (78.17%) |
| 3R5 | 56,220,732 | 5.68 Gb | 48.84% | 56,134,324 | 5.66 Gb | 48.88% | 45,706,102 (81.42%) | 41,975,726 (74.78%) |
| 0MH1 | 48,991,552 | 4.95 Gb | 49.40% | 48,898,956 | 4.93 Gb | 49.46% | 42,107,603 (86.11%) | 39,291,598 (80.35%) |
| 0MH2 | 49,655,044 | 5.02 Gb | 49.80% | 49,561,090 | 4.99 Gb | 49.86% | 43,401,965 (87.57%) | 40,701,106 (82.12%) |
| 0MH3 | 49,074,746 | 4.96 Gb | 49.64% | 48,983,736 | 4.94 Gb | 49.71% | 42,428,887 (86.62%) | 39,684,412 (81.02%) |
| 0MH4 | 51,672,582 | 5.22 Gb | 49.91% | 51,577,354 | 5.20 Gb | 49.97% | 44,010,688 (85.33%) | 40,960,844 (79.42%) |
| 0MH5 | 47,784,104 | 4.83 Gb | 50.19% | 47,695,816 | 4.81 Gb | 50.25% | 40,408,323 (84.72%) | 37,623,236 (78.88%) |
| OC1 | 54,254,678 | 5.48 Gb | 49.90% | 54,173,556 | 5.46 Gb | 49.95% | 45,758,834 (84.47%) | 42,517,434 (78.48%) |
| OC2 | 59,414,152 | 6.00 Gb | 49.47% | 59,308,182 | 5.98 Gb | 49.51% | 50,028,068 (84.35%) | 46,410,680 (78.25%) |
| OC3 | 57,315,416 | 5.79 Gb | 49.68% | 57,218,154 | 5.77 Gb | 49.72% | 47,591,512 (83.18%) | 44,113,078 (77.10%) |
| OC4 | 51,128,528 | 5.16 Gb | 50.12% | 51,039,316 | 5.14 Gb | 50.15% | 42,627,937 (83.52%) | 39,605,274 (77.60%) |
| OC5 | 45,551,858 | 4.60 Gb | 49.75% | 45,464,154 | 4.58 Gb | 49.78% | 37,518,090 (82.52%) | 34,768,716 (76.48%) |
| OH1 | 52,291,406 | 5.28 Gb | 50.15% | 52,187,588 | 5.26 Gb | 50.21% | 45,023,946 (86.27%) | 42,163,858 (80.79%) |
| OH2 | 47,829,842 | 4.83 Gb | 49.57% | 47,750,660 | 4.81 Gb | 49.63% | 41,361,214 (86.62%) | 38,712,786 (81.07%) |
| OH3 | 47,908,140 | 4.84 Gb | 49.69% | 47,822,552 | 4.82 Gb | 49.75% | 41,032,814 (85.80%) | 38,097,916 (79.67%) |
| OH4 | 51,504,146 | 5.20 Gb | 49.52% | 51,409,836 | 5.18 Gb | 49.58% | 44,480,007 (86.52%) | 41,645,100 (81.01%) |
| OH5 | 59,021,188 | 5.96 Gb | 49.57% | 58,916,342 | 5.94 Gb | 49.61% | 51,979,420 (88.23%) | 48,547,684 (82.40%) |
